# The CRF system and social behavior: a review

**DOI:** 10.3389/fnins.2013.00092

**Published:** 2013-05-31

**Authors:** Caroline M. Hostetler, Andrey E. Ryabinin

**Affiliations:** Department of Behavioral Neuroscience, Oregon Health and Science UniversityPortland, OR, USA

**Keywords:** CRF, urocortins, social behavior, stress, psychological, isolation, maternal behavior

## Abstract

The corticotropin-releasing factor (CRF) system plays a key role in a diversity of behaviors accompanying stress, anxiety and depression. There is also substantial research on relationships between social behaviors and the CRF system in a variety of taxa including fish, birds, rodents, and primates. Some of these relationships are due to the broad role of CRF and urocortins in stress and anxiety, but these peptides also modulate social behavior specifically. For example, the social interaction (SI) test is often used to measure anxiety-like behavior. Many components of the CRF system including CRF, urocortin1, and the R1 receptor have been implicated in SI, via general effects on anxiety as well as specific effects depending on the brain region. The CRF system is also highly responsive to chronic social stressors such as social defeat and isolation. Animals exposed to these stressors display a number of anxiety- and stress-related behaviors, accompanied by changes in specific components the CRF system. Although the primary focus of CRF research on social behavior has been on the deleterious effects of social stress, there are also insights on a role for CRF and urocortins in prosocial and affiliative behaviors. The CRF system has been implicated in parental care, maternal defense, sexual behavior, and pair bonding. Species differences in the ligands and CRF receptors have been observed in vole and bird species differing in social behavior. Exogenous administration of CRF facilitates partner preference formation in monogamous male prairie voles, and these effects are dependent on both the CRF R1 and R2 receptors. These findings are particularly interesting as studies have also implicated the CRF and urocortins in social memory. With the rapid progress of social neuroscience and in understanding the complex structure of the CRF system, the next challenge is in parsing the exact contribution of individual components of this system to specific social behaviors.

## Introduction

Many converging lines of evidence implicate the corticotropin-releasing factor (CRF) system in social behavior. The CRF system may affect social behaviors via a broader role in stress, anxiety, depression, and adaptation (Dunn and Berridge, 1990; Heinrichs et al., 1995; Arborelius et al., 1999; Koob and Heinrichs, 1999; Radulovic et al., 1999; Bale and Vale, 2004; Ryabinin et al., 2012), but there is also support that the CRF system specifically modulates social behaviors. This paper will provide a comprehensive review of existing research on this interaction, and aims to identify key areas for future research. This is particularly timely as social models are becoming increasingly sophisticated, our understanding of the complexity of the CRF system has evolved substantially, and the contribution of the CRF system to these behaviors has gained significant appreciation.

Although many of the effects of CRF on behavior are due to its role in initiating the hypothalamic-pituitary-adrenal (HPA) response to stressors, the CRF system throughout the rest of the brain is quite complex. This review will focus on the role of central CRF systems. Studies examining CRF's role in HPA functioning will primarily be limited to those that included brain measures and behavioral roles of the CRF system. The CRF system consists of two receptor subtypes, R1 and R2, and five ligands: CRF, Urocortin1 (Ucn1), Ucn2, Ucn3, and CRF-binding protein (CRFBP) (Vale et al., 1981; Behan et al., 1995; Steckler and Holsboer, 1999; Ryabinin et al., 2002; Fekete and Zorrilla, 2007). CRF and each of the urocortins have distinct distributions and binding affinities for each receptor and CRFBP, which lends this system to a high degree of complexity and behavioral specificity. Because of this complexity in receptors and ligands, an important caveat to the interpretation of pharmacological studies is that the effects of administration of agonists or antagonists do not implicate just one endogenous ligand or receptor.

In addition to a high concentration in the paraventricular nucleus (PVN) of the hypothalamus, CRF is abundant in the central amygdala (CeA) and hindbrain in mammals (Merchenthaler et al., 1982). CRF has a high affinity for the R1 receptor and CRFBP. Ucn1 is concentrated primarily in the centrally-projecting Edinger–Westphal nucleus, and to a lesser extent in supraoptic nucleus and dorsal lateral lemniscus, and binds with high affinity to R1 and R2 receptors, as well as CRFBP (Vaughan et al., 1995; Bittencourt et al., 1999; Fekete and Zorrilla, 2007). Ucn2 is produced in the PVN, other hypothalamic nuclei and the locus coeruleus, and binds primarily to the R2 receptor (Reyes et al., 2001; Yamauchi et al., 2005; Fekete and Zorrilla, 2007). Ucn3 is primarily found in hypothalamic and amygdalar regions, and has exclusive affinity for the R2 receptor (Lewis et al., 2001; Li et al., 2002; Fekete and Zorrilla, 2007). The R1 receptor is more abundant in the central nervous system than the R2 receptor, and is found in regions throughout the brain, including the olfactory bulb, cortex, septum, hippocampus, amygdala, and cerebellum (Bale and Vale, 2004; Fekete and Zorrilla, 2007). The R2 receptor is more restricted, concentrated primarily in the lateral septum, hypothalamus, dorsal raphe (DRN) and few other areas (Bale and Vale, 2004; Fekete and Zorrilla, 2007). The glycoprotein CRFBP is located throughout the brain, particularly in cortical regions, the amygdala, the bed nucleus of the stria terminalis (BNST), hypothalamus, and raphe nuclei (Potter et al., 1992). This distribution across many brain areas clearly indicates that the CRF system functions outside the classical HPA axis.

Understanding the role of this system outside of the stress response can be aided by examining how the CRF system has evolved. Despite the frequent assumption that glucocorticoid release is the primary outcome of CRF system activation, this response to stress emerged only during vertebrate evolution (Campbell et al., 2004b). In contrast, CRF/Ucn-like peptides appeared as early as mollusks and insects. Presumed functions for these homologous peptides in invertebrates include osmoregulation and feeding. The divergence of CRF/Ucn-like peptides occurred during vertebrate evolution, such that only one homologous peptide was found in tunicates (early vertebrates). Only thereafter did two gene duplications occur, first giving rise to segregation into CRF/Ucn1 and Ucn2/Ucn3 paralogs, and then into CRF, Ucn1, Ucn2, and Ucn3. Most likely, an additional duplication of Ucn1 occurred in amphibians, leading to sauvagine (Lovejoy, 2009; Lovejoy and Barsyte-Lovejoy, 2010).

Interestingly, the vertebrate CRF appears to be most distant from the ancestral peptides. Through its specialization for regulation of the HPA axis, it has lost many of its early characteristics (Coast, 1998; Lovejoy, 2009). Therefore, considering CRF as the prototypical CRF receptor ligand can be misleading. In parallel to the CRF/Ucn peptides, the genes homologous to the two CRF receptors and CRFBPs are also found in insects (Chang and Hsu, 2004; Huising and Flik, 2005). The link between CRF/Ucns and the stress response emerged only in vertebrates. Yet even in vertebrates, these peptides are involved in non-overlapping functions between taxa, such as metamorphosis in amphibians, and osmoregulation (via the urophysis) in fish. In this regard, it is worth noting that genetic deletion of either Ucn2 or Ucn3 in mice does not produce overt effects on stress reactivity, but does alter social behaviors, suggesting that mammals have adapted some of the CRF-related peptides to specific regulation of social interactions (SIs) (Deussing et al., 2003; Breu et al., 2012).

The CRF system has been studied in social contexts in a variety of taxa including fish, birds, rodents, and primates. The majority of research on the CRF system and social behaviors is in mammals, particularly rodents, therefore this review will focus on them. Short excursions into other taxa will be included where comparisons are illuminating.

## Developmental and adult effects of social housing conditions on CRF

### Immediate effects of CRF on isolation response

It has been demonstrated in many species that separation from the mother leads to an immediate increase in distress vocalizations from infants. Endogenous increases in central CRF following isolation (Walker et al., 1991) are thought to inhibit vocalizations. Cortisol (or corticosterone) response tends to peak during the same time period that vocalizations decline, ~1 h after separation onset. Exogenous administration of CRF inhibits separation-induced vocalizations in rats (Insel and Harbaugh, 1989; Harvey and Hennessy, 1995), guinea pigs (Hennessy et al., 1991; Hennessy, 1997), and Japanese quail (Launay et al., 1993). These vocalizations are blocked or reversed with pre-treatment with a CRF-receptor antagonist (Hennessy et al., 1992; Harvey and Hennessy, 1995; McInturf and Hennessy, 1996), indicating that separation-induced vocalizations are mediated by CRF receptors. Although the site of action is unknown, these effects are independent of CRF's actions on HPA activity (Hennessy, 1997).

### Response of CRF system to early isolation and separation

The early neonatal period in rodents is characterized by a “stress hyporesponsive period” (SHRP), during which pups show blunted HPA response to stressors (Levine, 2001). The SHRP is dynamically regulated by the dam, with different aspects of her care acting on specific aspects of the stress response. For example, tactile stimulation regulates changes in the brain associated with maternal deprivation, feeding regulates adrenal responsivity, and passive physical contact suppresses the stress response itself [as reviewed in Levine (2001)]. The SHRP is absent in maternally deprived pups. Loss of this regulating maternal influence early in life has significant consequences for the development of the HPA axis, including CRF gene expression in the hypothalamus, and related behaviors.

The handling procedure examines the effects of short-term (15 min) repeated maternal separations (MSs) on offspring. In the wild, dams must periodically leave the nest for short periods (for example, to forage for food). Therefore, handling is thought to better model naturalistic conditions than typical laboratory rearing under which pups have continuous access to the dam. Indeed, un-disturbed control groups tend to show HPA physiology and (hyper-) responsivity more similar to maternally deprived rats (see below) than handled animals (Plotsky and Meaney, 1993; Plotsky et al., 2005). Compared to un-handled rats, animals receiving early handling experience show reduced novelty-induced fear (Bodnoff et al., 1987), stress-induced anxiety (Meerlo et al., 1999), predator-induced behavioral inhibition (Padoin et al., 2001), and increased activity in the open field (Levine, 1967). Handled rats also show reduced HPA response to stress, as evidence by a lower endocrine response (plasma ACTH and corticosterone) to stressors, as well as a more rapid return to baseline at the end of the stressor (Plotsky and Meaney, 1993).

The effects of handling are observed within only a few days. Compared to undisturbed controls, increases in CeA CRF mRNA emerge only after 4 days of handling (Fenoglio et al., 2004), and with 1 week of handling CRF mRNA is elevated in the CeA and BNST, and decreased in the PVN (Fenoglio et al., 2004). When tested as 3–4-month-old adults, male rats that had received neonatal handling had lower levels of CRF mRNA in the PVN, CeA, BNST, and locus coruleus/parabrachial nucleus (LC/PBN), as well as reduced CRF protein in the PVN, CeA, BNST, LC/PBN, and median eminence than undisturbed animals (Plotsky and Meaney, 1993; Viau et al., 1993; Plotsky et al., 2005). Clearly, this modest but naturalistic procedure has profound effects on the CRF system.

Short-term separation (i.e., handling) produces less stress-reactive offspring, but longer term repeated separations (of many hours) can have the opposite effect. This effect of MS has been studied in a diversity of species and across many developmental timepoints. The stress of separation itself likely has direct effects on the pup, but the effects of MS are also mediated by changes in maternal care due to separation. When dams are given access to a foster litter during separation (rather than isolation, as is the common method), the effects of the MS procedure are attenuated or even prevented entirely (Huot et al., 2004). Although often not considered, this study demonstrates that even a simple procedure may have multiple effects on the social environment that each contribute to the end phenotype.

As with handling, the effects of MS are rapid. Maternal deprivation in preweanling female sheep decreases CRF-immunoreactivity (-ir) nerve terminals in the median eminence after only 3 days (Wankowska et al., 2006). Daily MS over the first 2 weeks of life increases CRF-R1 in the PVN of female juvenile rats (Rees et al., 2008). Interestingly, early isolation in domestic piglets may have very delayed consequences on CRF. In one study (Kanitz et al., 2004), animals were isolated for 2 h daily from postnatal day (PND) 3–11, and CRF-ir was investigated using radioimmunoassay from animals shortly after isolation (PND 12) and after weaning (PND 56). MS had no effect of on CRF-ir in the PND 12 animals, but at 56 days old, animals that had received early repeated isolation had higher CRF-ir in the amygdala, and reduced CRF-ir in the hypothalamus. This finding highlights the need for studies on long-term consequences of early social stressors, as immediate effects of even repeated social stressors may provide an incomplete picture. The effects of deprivation persist well into adulthood, as daily MS during the pre-weaning period increases CRF receptor binding in the raphe nucleus and CRF-ir in the median eminence and PBN of 108-day-old male rats, but no difference in other cortical or limbic regions examined (Ladd et al., 1996).

Babygirija et al. (2012) examined the post weaning social environment on adult behavior and physiology of rats exposed to MS from PND 2–14. These rats were placed in post-weaning social housing either with other MS-reared rats or control (non-MS reared) rats. As expected, MS increased CRF mRNA in the PVN of adult rats, but this was attenuated when rats were housed with control animals. Therefore, the effects of early isolation in male rats are strongly influenced by later social experience (Babygirija et al., 2012). Seemingly paradoxically, these same MS rats housed with control partners have higher CRF cell counts in the PVN following a restraint stress than animals housed with similarly treated peers.

Traditional laboratory species, like most mammals, rely primarily on maternal care during postnatal development. Studies in biparental species, such as degus and prairie voles, indicate that paternal influences are also critical for the development of the CRF system. The degu (*Octodon degu*) is a caviomorph rodent that differs from typical laboratory rodents in a number of ways. Although the weaning age of degus is quite similar to other laboratory rodent species (~PND 21), degus are born highly precocial following a 90–100 day gestation. The infant degu brain is more developed at birth and postnatal brain development is slower than a rat or mouse. In this species, 1 h of daily parental deprivation increases CRF-ir in the BLA, but decreases CRF-ir in the CeA, hippocampus, and somatosensory cortex, without altering PVN CRF-ir (Becker et al., 2007). Moreover, complete removal of only the father is associated with more CRF positive cells in the orbitofrontal cortex and BLA, but lower numbers of CRF positive cells in hippocampal regions at weaning (Seidel et al., 2011). However, by adulthood the only observed difference was a decrease in CRF positive cells in the stratum pyramidale of the CA1 region (Seidel et al., 2011).

Prairie voles (*Microtus ochrogaster*) are a social monogamous rodent that typically rears offspring biparentally. In a study of adult voles that had been reared only by their mother vs. both parents, Ahern and Young (2009) found that R2 binding in the DRN was increased in mother-reared offspring compared to biparentally-reared offspring. Further, the R2 binding in this area was positively correlated with the amount of licking and grooming in the natal nest, suggesting a direct link between parental behavior and the R2 receptor in DRN.

### Response of CRF system to isolation over adolescence

McCormick et al. (2007) looked at both short- and long-term responses of the PVN and CeA to different social stressors in adolescent rats. Brief (1 h) exposure to isolation induces a typical acute stress response: CRF mRNA is increased in the PVN but remains unchanged in the CeA. In males, this increase in the PVN is maintained if they are subsequently housed with a novel social partner, but is attenuated when they are returned to a familiar cagemate. Housing with a novel social partner after isolation also increases CRF mRNA in the CeA of males only. Isolation-induced changes in females were not affected by the post-isolation social environment, consistent with other research demonstrating greater sensitivity of the CRF system of males to many social stressors.

To investigate long-term changes in CRF mRNA to different social stressors, juvenile rats were isolated for 1 h daily over 2 weeks (McCormick et al., 2007). Animals were then either returned to their homecage with a familiar cagemate, or were housed with a different unfamiliar cagemate each day. Control rats were continuously housed with a familiar cagemate. Repeatedly-isolated animals had higher levels of CRF mRNA in the PVN, and showed habituation to an additional isolation (i.e., no change in CRF). Control males showed an increase in CRF mRNA in the CeA following acute isolation stress, but not the other groups. Animals that were briefly isolated and exposed to a new partner every day had overall higher levels of CRF mRNA in this region than animals that were only isolated daily (McCormick et al., 2007). Similar to a lack of effects of acute social stress, female rats did not differ on either baseline or isolation-induced CRF in the CeA.

Juvenile rats that are housed in isolation following weaning have an increase in R2 in the DRN, and this is associated with decreased sensitivity to CRF-induced release of 5HT into the nucleus accumbens (Lukkes et al., 2009b). This increase in R2 in the DRN may promote isolation-induced changes in social anxiety (Lukkes et al., 2009a). Blockade of R2, but not R1 receptors in the DRN reversed the anxiogenic effects of isolation on plus maze behavior (Bledsoe et al., 2011). It should be noted that similar increases in R2 were observed in prairie voles reared without a father (Ahern and Young, 2009), as discussed above.

Male and female prairie voles that have been each short-term (4 day) and long-term (21 day) isolated following weaning show an increase in CRF-ir in the PVN and an accompanying increase in peripheral corticosterone (Ruscio et al., 2007). It is interesting to note that there is no difference between animals that are housed with a familiar same-sex sibling compared to those that are placed with a novel same-sex partner (Ruscio et al., 2007). Similarly, 6 weeks of post-weaning isolation in male prairie voles increases CRF mRNA in the PVN (Pan et al., 2009).

Male R2 knock out (KO) mice show greater behavioral sensitivity to post-weaning isolation housing than their wild-type littermates (Gresack et al., 2010). Isolated animals show increased exploratory rearing when initially placed in a novel environment (a locomotor activity chamber), and this is independent of genotype. However, isolated R2 KO's demonstrate poor habituation to the chamber, characterized by elevated activity and hole-poking compared to controls (Gresack et al., 2010). There are no behavioral differences in the locomotor activity between socially-housed KOs and wild-type mice. This supports the idea that R2 receptors are important for habituation to stressors, including social stressors such as isolation.

Domestic chickens (*Gallus gallus*) are a precocial bird species that do not receive postnatal care. Daily social isolation from conspecifics over PND 4–26 does not affect basal expression of R1 mRNA, but exposure to a novel restraint stress increases R1 mRNA in the thalamus/hypothalamus of male chickens that have undergone isolation (Goerlich et al., 2012). Females showed no upregulation of R1 mRNA, regardless of early experience, which is in agreement with rodent findings that social isolation effects are more robust in males. Unfortunately, this study did not examine CRF R2 levels, which would have allowed further comparisons with rodent studies.

### Response of CRF system to adult isolation

There are limited available data on the role of the CRF system in response to acute isolation in adults. Oral administration of the R1 antagonist antalarmin in marmosets increased arousal response to acute separation from a pairmate (French et al., 2007). Studies examining the effects of acute, repeated, and chronic social isolation in adult prairie voles have focused on alterations of hypothalamic and hippocampal CRF measures. Specifically, exposure to either short-term (1 h) acute or daily repeated isolation increases CRF mRNA in the hypothalamus and hippocampus, but not in animals that are chronically isolated for 4 weeks (Pournajafi-Nazarloo et al., 2009, 2011). In contrast, chronic, but not acute or repeated, isolation leads to an increase in R2 mRNA in the hippocampus. All isolation stressors decreased R2 in the hypothalamus, and none had any effect on R1 receptors in either region (Pournajafi-Nazarloo et al., 2009, 2011). No sex differences were observed in any measures.

In another study in prairie voles, 4 weeks of isolation does not affect basal expression of CRF-ir in the PVN (Grippo et al., 2007b), however, there is an interesting sex difference in this region following resident-intruder challenge. Isolated females show a greater percentage of CRF cells in the PVN co-labeled with c-fos, but males show no difference based on housing history (Grippo et al., 2007b). Isolated females also have more CRF-positive cells in the PVN following resident-intruder stress [although this study did not examine males (Grippo et al., 2007a)]. These findings suggest that female prairie voles may show greater HPA sensitivity to isolation than males. This contrasts with studies of early isolation stress, which show higher sensitivity of male animals.

In a study of male voles, Bosch et al. (2009) looked at behavioral and physiological consequences of isolation from different types of social partners. Isolation from a female partner, but not a male sibling, increased passive stress-coping behavior in each the forced swim and tail suspension test. Interestingly, males that were housed with females had increased CRF mRNA in the medial BNST, and this was independent of whether they were subsequently isolated from their female partner (Bosch et al., 2009). The behavioral effects were blocked by central administration of a general CRF antagonist (d-Phe-CRF), as well as antagonists specific for each the R1 (CP154526) and R2 (Astressin-2B) receptors (Bosch et al., 2009). Lack of an effect of chronic isolation on CRF-ir in the PVN in voles (Grippo et al., 2007b) is contrasted by a study of California mice (*Peromyscus californicus*) in which long-term isolation in adulthood is associated with a reduction in basal CRF-ir in the PVN in males (Chauke et al., 2012). Similar to voles, these male California mice also show heightened expression of CRF and reduced neuronal activation in the CeA and PVN following a stressor (Chauke et al., 2012).

Although site-specific manipulation of CRF receptor activity in adult isolates has not been performed, we can speculate on candidate regions. Ehlers et al. (1993) examined changes in binding of ovine CRF (a R1 ligand with low affinity to CRF-BP) throughout the brain of adult rats that had been isolated for 6 weeks. Although no changes were observed in the hypothalamus, there was a decrease in the prefrontal cortex, and an increase in CRF binding in the cingulate cortex, piriform cortex, and cerebellum (Ehlers et al., 1993). Not surprisingly, these alterations were more pronounced in males.

It is pertinent to note that many studies in behavioral neuroscience use isolated subjects due to methodological simplicity, without addressing the direct and confounding effects this housing condition may have on results. Many of the common laboratory models are highly social species (rats, mice, primates), and group housing is often more naturalistic. As reviewed here, chronic and repeated isolation are significant stressors for these animals, with long-term consequences on the CRF system and we caution against over-interpretation of studies involving only isolated subjects. For example, Lodge and Lawrence (2003) investigated the effects of the R1 antagonist antalarmin on alcohol self-administration in rats, but all subjects were reared in isolation. Therefore, it is difficult to know if the ability of antalarmin to reduce ethanol self-administration is significantly influenced by social stress effects on central CRF systems. As the CRF system has been proposed as a therapeutic target for a number of stress- and anxiety-related disorders (Kehne and Cain, 2010; Edwards et al., 2011; Paez-Pereda et al., 2011), it is critical that our behavioral models address or control for such isolation effects.

## Anxiety/social interaction

The CRF system is broadly implicated in anxiety across a number of behavioral paradigms, including open field, elevated plus maze, light-dark box, defensive withdrawal, and the SI test (File and Hyde, 1978). In the SI test, an animal is exposed to a novel conspecific and the time spent engaging in active social contact is recorded. Although this may differ based on laboratory, the active social contact measure includes all SIs, including sniffing, grooming, following, mounting, kicking, boxing, wrestling, and crawling, as a single measure of SI. Passive social contact, in which animals are touching but not interacting, is a separate measure. Using this metric, SI is inversely associated with anxiety, such that decreased levels of SI are indicative of anxiogenic state. Although rarely considered, the SI test may also measure direct effects on social behavior, as suggested by studies showing dissociated behavioral effects of CRF manipulations on SI and other anxiety measures (Zhao et al., 2007; Lee et al., 2008; Breu et al., 2012).

Central administration of CRF, Ucn1, or the R1 agonist stressin1-A decreases SI in rats (Dunn and File, 1987; Sajdyk et al., 1999; Campbell et al., 2004a; Gehlert et al., 2005; Zhao et al., 2007). A general CRF receptor or R1 specific antagonist has no direct effects on SI, but each are effective at blocking the anxiogenic effects of Ucn1 and CRF when administered as a pre-treatment (Sajdyk and Gehlert, 2000; Campbell et al., 2004a; Gehlert et al., 2005). Priming with a sub threshold dose of either Ucn1 (BLA: Sajdyk et al., 1999; Donner et al., 2012); (BNST: Lee et al., 2008) or CRF (BLA: Sajdyk et al., 1999) decreases SI and these behavioral effects can be observed for up to 4 weeks post-injection (Lee et al., 2008). These priming effects can be blocked by co-administration of an R1 antagonist (Sajdyk and Gehlert, 2000; Lee et al., 2008).

Each Ucn1 and CRF decreases SI when injected directly into the BLA (Sajdyk et al., 1999; Gehlert et al., 2005; Spiga et al., 2006). The behavioral specificity of Ucn1 on social anxiety is site-specific as administration into the BLA is anxiogenic across different behavioral and physiological markers (Sajdyk et al., 1999), however, it is highly specific for social anxiety in the BNST (Lee et al., 2008), and has no effect on any measure of anxiety when administered into the nucleus accumbens (Lee et al., 2008). Overall, these lines of evidence strongly implicate the R1 receptor and CRF or/and Ucn1 as modulating SI, consistent with their anxiogenic role in other tests.

There are limited data regarding the roles of Ucn2 and Ucn3 on SI. Male Ucn2 KO mice spend more time in passive social contact than wild-types, but do not differ in active SI (Breu et al., 2012). These KO males are also less aggressive. Females, however, do not differ in either measure in the SI test. Urocortin 3 has no effect on SI when administered i.c.v. (Zhao et al., 2007). At present, the role of R2 and its selective ligands (Ucn2 and Ucn3) in SI remain underexplored.

## Social defeat stress

Exposure to an aggressive conspecific induces rapid behavioral and neural adaptations. This social defeat stress (SDS) is an ethologically relevant model of stress, and demonstrates strong translational and construct validity (Wood et al., 2012). In this procedure, individuals are placed into the home cage of a larger, dominant, aggressive male. The resident male will attack and defeat the intruder male within 2 min, after which the subject is usually placed behind a mesh screen to protect from further attack, while maintaining the social stressor. The effects of the SDS on the CRF system are immediately apparent. In response to a single exposure, SDS induces an increase of CRF mRNA in the BNST and CeA, but not the PVN in rats (Funk et al., 2006), and activates CRF R2-positive neurons in the medial amygdala (Fekete et al., 2009). Six hours following defeat, CRF-ir is decreased in the hippocampus (Panksepp et al., 2007).

A single social defeat leads to an immediate anxiogenic effect, as demonstrated by a reduction in open arm time on the elevated plus maze (Heinrichs et al., 1992). The expression of SDS-induced anxiety is blocked by administration of the general CRF antagonist α-helical CRF into the CeA prior to elevated plus maze testing (Heinrichs et al., 1992). Liebsch et al. (1995) examined the effects of an antisense oligodeoxynucleotide blocking R1 mRNA in the CeA. Animals treated with the antisense oligodeoxynucleotide for 4 days prior to SDS testing showed greater open arm activity following the SDS compared to controls, indicating anxiolytic effect of this treatment (Liebsch et al., 1995).

SDS also leads to a rapid development of submissive and defensive behaviors in response to non-threatening social stimuli. This has been best characterized in male Syrian hamsters (*Mesocricetus auratus*), a species which typically demonstrates high levels of territorial aggression but switches to a defensive strategy following a single SDS (Huhman et al., 2003). The role of the CRF system in each the acquisition and expression of this conditioned social defeat (CSD) behavior has been examined. In order to study the role of the CRF system in the acquisition of CSD, Cooper and Huhman (2007, 2010) examined the effects of CRF antagonists administered prior to SDS on later CSD behavior. The non-selective antagonist D-Phe CRF and R2 specific antagonist anti-SVG-30 both reduced submissive/defensive behavior when administered i.c.v. (Cooper and Huhman, 2010), but the R1 antagonist CP154526 had no effect on later behavior. When administered directly into the DRN, D-Phe CRF reduced both submissive/defensive and social behaviors following SDS, but had no effect on controls (Cooper and Huhman, 2007). Intra-DRN administration of anti-SVG-30 also increased social behavior, but had no effect on submissive/defensive behavior. Taken together, these findings implicate the R2 receptor and urocortins in the acquisition of conditioned defeat, with R1 receptors playing a negligible role. However, intra-amygdalar antalarmin, an R1 antagonist, prevents increases in submissive/defensive behaviors when administered immediately following SDS in mice (Robison et al., 2004).

The R2 receptor is also implicated in the expression of CSD in hamsters. The non-specific CRF receptor antagonist D-Phe CRF selectively decreases the expression of submissive/defensive behavior when administered into the lateral ventricles, BNST, and DRN, but not the CeA (Jasnow et al., 1999, 2004; Cooper and Huhman, 2007). CRF receptor blockade in the DRN reduces both the acquisition and expression of CSD (Cooper and Huhman, 2007). Similarly, the R2 antagonist anti-SVG-30 reduces CSD of animals treated directly into the ventricles, BNST, or DRN (Cooper and Huhman, 2005, 2007). In contrast to a general antagonist, anti-SVG-30 in the DRN is specifically reduces expression, but not acquisition, of CSD (Cooper and Huhman, 2007). Peripheral (i.p.) or central (i.c.v.) blockade of R1 receptors with the antagonist CP154526 has no effect on CSD (Jasnow et al., 1999; Cooper and Huhman, 2005).

Repeated or chronic SDS has even more profound effects, including reduction in body and adrenal weight, increases in depressive-like behavior, and changes in autonomic function (Wood et al., 2012). These effects are blocked by daily treatment with the R1 antagonist NBI-30775 over the course of chronic social defeat in rats (Wood et al., 2012). Additionally, NBI-30775 treated animals demonstrated a more active and less defensive response during the SDS, suggesting that R1 receptors may play a role in acute response to this social stressor (Wood et al., 2012). This is further supported with a study of transgenic mice expressing conditional, postnatal forebrain R1 deficiency (Wang et al., 2011). These mice are protected against chronic social defeat-induced impairments in object recognition, spatial memory, and hippocampal remodeling (Wang et al., 2011).

A naturalistic model of chronic social defeat has been examined by placing a mixed-sex group of rats in a visible burrow system (VBS). Dominance hierarchies are quickly established, with a single dominant male and three subordinate males. Subordinates display a number of behavioral and physiological characteristics typical of chronically stressed animals. Interestingly, a subgroup of subordinates fail to show a corticosterone increase following a novel stressor, and there is evidence that these non-responders also differ in CRF regulation of the HPA axis. Specifically, although dominant rats and typical subordinate rats show similar levels of CRF mRNA and cell numbers in the PVN to each other (Albeck et al., 1997; Choi et al., 2006), non-responding subordinates have reduced levels (Albeck et al., 1997). However, CRF mRNA is increased in the CeA of subordinates, regardless of their stress response (Albeck et al., 1997). It must be noted that another study found no difference in CeA CRF mRNA between subordinate and dominant animals (Choi et al., 2006), although this may reflect methodological differences between the two studies. Relative to both control and dominant rats, subordinates also have elevated CRF mRNA in BNST (Choi et al., 2006). Although there are inconsistencies between studies, both implicate the extended amygdala as a target of VBS stress on the CRF system.

These studies on social stress and the CRF system warrant some methodological considerations. All of the experiments discussed in this section specifically examine males. Although isolation and SDS are not as stressful for females, females do show a robust chronic stress phenotype in response to social instability (Haller et al., 1999; Schmidt et al., 2010). Examining the effects of an appropriate social stressor, such as group instability, in females is a critical avenue for this field. Additionally, many studies use animals that are housed in isolation, which may influence CRF systems (see above). Some studies even neglect to describe housing conditions at all. Finally, stress control conditions also vary, ranging from no stress, handling, or exposure to a novel but non-aggressive male. These different behavioral conditions likely have their own effects on the CRF system. A summary of these experimental conditions, and others, are described in Table 1.

**Table 1:** **Experimental conditions for studies on the role of the CRF system in social defeat stress**.

**Study**	**Species (strain)**	**Sex**	**Housing**	**Control condition**	**Duration of stressor**	**Measure/manipulation**	**Site/route**
**EFFECTS OF SOCIAL DEFEAT STRESS ON CENTRAL CRF SYSTEM**							
Funk et al., 2006	Rat (Wistar)	Male	Not spec.	No stress	30 min	CRF mRNA (*in situ*)	
Panksepp et al., 2007	Rat (Long–Evans)	Male	Group	Novel non-aggressive	30 min	CRF-like peptide (RIA)	
Fekete et al., 2009	Rat (Wistar)	Male	Single	Handling only	75 min	CRF R2 mRNA *in situ* and c-fos ICC	
**PHARMACOLOGICAL EFFECTS ON THE EXPRESSION AND ACQUISITION OF CONDITIONED DEFEAT STRESS**							
Robison et al., 2004	Mice (C57BL/6)	Not spec.	Single	Antalarmin, non-stress	10 min	Antalarmin	BLA
Jasnow et al., 1999	Syrian hamsters	Male	Single	None	4 × 5 min over 1 day	CP-154,526	i.p.
	Syrian hamsters	Male	Single	None	4 × 5 min over 1 day	D-Phe CRF	i.c.v.
Jasnow et al., 2004	Syrian hamsters	Male	Single	None	15 min	D-Phe CRF	BNST
	Syrian hamsters	Male	Single	None	15 min	D-Phe CRF	CeA
Cooper and Huhman, 2005	Syrian hamsters	Male	Single	Empty resident cage	15 min	anti-SVG-30	i.c.v.
	Syrian hamsters	Male	Single	Empty resident cage	15 min	CP-154,526	i.c.v.
	Syrian hamsters	Male	Single	Empty resident cage	15 min	anti-SVG-30	BNST
Cooper and Huhman, 2007	Syrian hamsters	Male	Single	Empty resident cage	15 min	D-Phe CRF	DRN
	Syrian hamsters	Male	Single	Empty resident cage	15 min	anti-SVG-30	DRN
	Syrian hamsters	Male	Single	Empty resident cage	15 min	D-Phe CRF	DRN
	Syrian hamsters	Male	Single	Empty resident cage	15 min	anti-SVG-30	DRN
Cooper and Huhman, 2010	Syrian hamsters	Male	Single	None	15 min	D-Phe CRF	i.c.v.
	Syrian hamsters	Male	Single	None	15 min	anti-SVG-30	i.c.v.
	Syrian hamsters	Male	Single	None	15 min	CP-154,526	i.c.v.
**PHARMACOLOGICAL EFFECTS ON OTHER BEHAVIORAL CONSEQUENCES OF SOCIAL DEFEAT STRESS**							
Heinrichs et al., 1992	Rat (Wistar)	Male	Not spec.	No stress	30 min	α-helical CRF	i.c.v.
	Rat (Wistar)	Male	Not spec.	No stress	30 min	α-helical CRF	CeA
Liebsch et al., 1995	Rat (Wistar)	Male	SINGLE	None	10 min	Antisense oligonucleotide	CeA
Wood et al., 2012	Rat (Sprague Dawley)	Male	SINGLE	Novel cage	30 min × 7 days	NBI-30775	s.c.
Wang et al., 2011	Mice	Male	SINGLE	No stress	24 h × 21 days	CRH R1 deficiency	Forebrain

Housing indicates how animals were housed prior to any SDS testing (“not spec.” indicates that pre-testing housing was not indicated, and is presumed to be isolation). Control condition refers specifically to any behavioral controls (“none” indicates all animals were tested for response to stress).

## Social stress in fish

The involvement of the CRF system in social stress in mammals (reviewed above), warrants comparison to evolutionary earlier taxa, such as fish. The CRF system of fish is quite similar to that of mammals, including CRF, Ucn1, Ucn2, Ucn3, CRFBP and both receptor subtypes. However, cortisol release is stimulated via two mechanisms: the hypothalamic-pituitary-interrenal (HPI) axis and the caudal neurosecretory system (CNSS). The HPI axis is similar to the mammalian HPA axis: CRF is released from the preoptic nucleus (POA) of the hypothalamus (analogous to the mammalian PVN) to stimulate ACTH release from the pituitary. ACTH then stimulates cortisol release from interrenal cells. The CNSS, which lacks a mammalian homologue, is characterized by magnocellular Dahlgren cells located along terminal segments of the spinal cord (Lu et al., 2004; McCrohan et al., 2007). Dahlgren cells both produce and release CRF and Urotensin-I (UI; orthologous to mammalian Ucn1) and projects axons directly to the urophysis, a neurosecretory organ. The CNSS also lacks cortisol-driven negative feedback that is characteristic of the HPA and HPI axis. Besides areas involved in regulation of these axes, CRF and urocortins are also expressed in extra-hypothalamic areas (Lovejoy and Balment, 1999; Alderman and Bernier, 2007).

Many teleost fish form dominance hierarchies, with dominant and subordinate individuals displaying different behavioral, endocrine, neurophysiological, and metabolic phenotypes. Specifically, dominant animals display higher levels of aggression, and subordinates show blunted growth, decreased access to food, and increased metabolic rate. In order to examine these phenotypes in the laboratory two size-matched, unfamiliar fish are placed in a tank. The animals are separated from each other by an opaque divider for a 1- to 3-day acclimation period. The divider is then removed and animals can directly interact. Dominance is established rapidly, and is determined by which animal in the dyad displays the most aggressive attacks, patrolling the water column, and feeding access.

Subordinate rainbow trout (*Oncorhynchus mykiss*) show hyperactive HPI activity, as measured by chronically elevated cortisol and ACTH (Sloman et al., 2001; Doyon et al., 2003; Alderman et al., 2008; Bernier et al., 2008; Jeffrey et al., 2012). This hyperactivation may be supported in part through elevated levels of CRF mRNA in the POA that are observed within a few hours of subordination (Bernier et al., 2008) and stay elevated for many days (Doyon et al., 2003; Bernier et al., 2008). Elevated cortisol levels are observed out to 7 days (Sloman et al., 2001), but CRF mRNA in the POA does not differ between dominant and subordinate animals after 5 days (Jeffrey et al., 2012). Similarly, whole brain CRF mRNA levels do not differ based on social status after 5 days of dyadic interaction in zebrafish (Pavlidis et al., 2011). It appears that heightened levels of cortisol do not habituate to subordination stress, whereas CRF mRNA in the POA habituates within a few days to basal levels (Table 2).

**Table 2:** **Relative cortisol and mRNA levels of subordinate trout to dominant or control animals (n.d. = no difference)**.

**Stressor**	**Time**	**Cortisol**	**POA**			**CNSS**	
			**CRF**	**UI**	**CRFBP**	**CRF**	**UI**
Subordination	8 h	↑^a^	↑^b^	n.d.^b^	n.d.^a^	n.d.^b^	n.d.^b^
	24 h	↑^a^,^b^	↑^b^	n.d.^b^	n.d.^a^	n.d.^b^	n.d.^b^
	3 d	↑^d^	↑^d^				
	5 d	↑^e^	n.d.^e^		n.d.^e^		
Isolation	4 h	↑^c^	n.d.^c^			n.d.^b^	n.d.^b^
	24 h	↑^b^,^c^	↑^b^,^c^	−^b^			
	3 d	n.d.^c^	n.d.^c^				
	4 d	n.d.^b^				↓^b^	↓^b^

a *Alderman et al., 2008*;

b *Bernier et al., 2008*;

c *Doyon et al., 2005*;

d *Doyon et al., 2003*;

e *Jeffrey et al., 2012*.

Visual cues from a dominant male are sufficient to cause transient changes in stress-related gene expression in an African cichlid, *Astatotilapia burtoni* (Chen and Fernald, 2011). In a variation of the social dominance test described above, male fish were housed with a female on one side of a divided tank. On the other side were a female and a larger, dominant male fish. Following a 2 day habituation period, the opaque divider was removed, and animals remained separated via a clear divider that allowed visual, but not physical or olfactory contact. Relative to control males, whole brain CRF and CRFBP were increased at 3 days of visual contact with a dominant male (Chen and Fernald, 2011). Similarly, CRF R2 mRNA was increased, but CRF R1 mRNA decreased, following 3 days of visual social stress. However, no differences were observed in any CRF system-related measures at either 1 day or 1 week of this social stress. These findings suggest that visual information during social stress is sufficient to induce broad changes in the CRF system, but may show a delayed response compared to physical interaction.

Likewise, exogenous administration of CRF may influence dyadic behavior in trout by influencing behavior directly. The effects of CRF on behaviors in a size-matched dyadic encounter are unclear. Fish treated with higher doses of i.c.v. CRF are more likely to become subordinate than controls (Backstrom et al., 2011). Central administration of CRF paradoxically decreases the number of both attacks and retreats, as well as the latency to attack (Carpenter et al., 2009). However, administration of UI, a general CRF antagonist (α-helical CRF) or the CRF R1 specific antagonist antalarmin had no effect on dyadic aggression (Backstrom et al., 2011). There is also evidence for differential neural response to different types of social stressors. Whereas cortisol remains elevated with prolonged subordination, isolation-induced levels of cortisol return to baseline within a few days (Doyon et al., 2005). Similarly, CRF mRNA in the POA seems to have both a delayed and more transient response to isolation (Doyon et al., 2005; Bernier et al., 2008; Table 2). The CNSS does not appear to be immediately responsive to subordination stress, but each CRF and UI mRNA is elevated in the CNSS following long-term isolation (Bernier et al., 2008). While these studies clearly show the greater involvement of the CRF than UI in regulation of social behavior in fish, the sparse analyses of other CRF R2 ligands makes it difficult to assess the role of urocortins in social behaviors.

## Individual differences in response to social stress

Individual differences in the response of the CRF system to social stress have been observed in a number of rodent models (Albeck et al., 1997; Elliott et al., 2010; Wood et al., 2010; De et al., 2011). There is also some evidence that individuals may be genetically susceptible to effects of early social environment on later behavior. Barr et al. (2009) identified a single nucleotide polymorphism (SNP) in the promoter region of the *crh* gene (which encodes CRF protein) of rhesus macaques. A gene-environment interaction was found between this SNP and rearing experience. Specifically, peer-reared monkeys that were heterozygous for the SNP showed significant decreased environmental exploration and increased voluntary ethanol consumption compared to mother-reared animals and homozygous peer-reared monkeys (Barr et al., 2009). Similarly, in human patients with a history of child sexual abuse, a haplotype of the CRF R1 gene protects against the development of alcohol abuse (Nelson et al., 2010). Understanding how genetic polymorphisms and epigenetic regulation of the CRF system affect response to social stress and social behavior directly is an exciting direction for future study.

## Other social stressors

Pheromones are by definition social signals, as release of a pheromone from one individual is specifically meant to elicit a response in another individual of the same species. The CRF system has been implicated in behavioral responses to alarm pheromone, which is released following footshock (Kikusui et al., 2001) and can increase stress- and anxiety-sensitive behaviors in exposed rats (Kiyokawa et al., 2005, 2006, 2007; Inagaki et al., 2008). Exposure to alarm pheromone affects male, but not female, sexual behavior in rats (Kobayashi et al., 2011, 2013). However, pre-treatment with the R1 antagonist CP154526 normalizes sexual behavior in male rats exposed to alarm pheromone (Kobayashi et al., 2011). Additionally, exposure to alarm pheromone increases the number of cells in the PVN double labeled for CRF and c-fos (Kobayashi et al., 2013). These findings suggest that alarm pheromone induces release of CRF from the PVN to modify sexual, and other behaviors, however, this was found in animals that were also tested for sexual behavior prior to tissue collection, so it should be interpreted cautiously. These studies clearly demonstrate behavioral responses to pheromones, a specifically social phenomenon, are mediated by the CRF system, in a sex-specific manner.

## Male aggression

Intermale aggression is often examined using the resident-intruder paradigm, in which an unfamiliar, non-aggressive intruder is placed in the home cage of the subject (Miczek, 1979). In these studies, the subjects are housed in isolation for an extended period of time prior to testing. Studies have implicated the CRF system in intermale aggression, although the exact mechanisms are unclear. Administration of CRF or its R2-acting paralogue sauvagine (i.c.v.) decreases aggression and sociability, while increasing defensive behaviors in mice (Mele et al., 1987). When tested in a novel arena, rather than home cage, CRF increased aggressive behavior in rats when administered into the amygdala (but not the highest dose), but not the lateral ventricle (Elkabir et al., 1990). Interestingly, i.c.v. administration of CRF decreased investigative behaviors, but at the highest dose in the amygdala promoted investigation (Elkabir et al., 1990). Male mice lacking Ucn2 show reduced aggression (Breu et al., 2012).

Contrasting effects were found in rats tested for stress-induced fighting. Stress-induced fighting consists of exposing a pair of rats to intermittent and inescapable foot shocks, with defensive aggression developing with repeated testing. Central administration of CRF facilitates aggressive behavior, and the antagonist α-helical CRF reduces stress-induced fighting (Tazi et al., 1987). The differences in effects observed in resident-intruder aggression and stress-induced fighting highlight the importance of dosage, site of administration, and behavioral conditions when comparing studies. This is further demonstrated in two studies of R2 KO mice. Using the resident-intruder test, Gammie et al. (2005) found no effect of R2 KO on aggression. However, when placed in a novel environment, R2 KOs show elevated levels of aggression (Coste et al., 2006).

Oral administration of the R1 antagonist SSR122343A reduces aggressive behavior in both hamsters and rhesus macaques (Habib et al., 2000; Farrokhi et al., 2004). However, KO of the R1 gene in mice has little effect on aggression. Specifically, the only behavioral difference in R1 KOs compared to their wild-type littermates is a reduced in offensive attacks to the belly area of an intruder (Gammie and Stevenson, 2006). There are also no differences in resident-intruder aggression in CRFBP KO mice (Gammie et al., 2008). Overall, although ligands of both CRF receptors are clearly involved in intermale aggression, their specific roles remain to be determined and are an open area for future research.

## Maternal aggression and parental behavior

Maternal defense in rodents is characterized by high levels of aggression toward a novel conspecific, and is often examined in the laboratory using a resident-intruder test. Pups are removed from the home cage during testing, and a novel, non-aggressive male intruder is placed in the cage. In contrast to intermale aggression, there is strong evidence for a major role of the CRF system in regulating maternal defense. Decreased CRF neurotransmission during lactation is thought to contribute to each the attenuated stress response in dams as well as promoting maternal aggression (Gammie et al., 2004; Chauke et al., 2012). This is supported by studies showing that central CRF administration into either the lateral ventricle or lateral septum reduces maternal aggression (Gammie et al., 2004; D'Anna and Gammie, 2009). Dams lacking CRFBP also show reduced aggressive behavior, but no difference in pup-directed maternal behaviors (Gammie et al., 2008). The CRF general antagonist D-Phe-CRF has no effect on aggression, although this may reflect a floor effect (Gammie et al., 2004). Administration of either Ucn1 or Ucn2 also inhibits maternal aggression (D'Anna et al., 2005). The ability of CRF and Ucn3 administered directly into the LS to decrease maternal aggression is blocked by co-administration of an R2, but not R1, antagonist (D'Anna and Gammie, 2009), implicating the CRF R2 receptor as a critical regulatory site for this behavior. This is supported by studies of R2 KO dams, which also show reduced maternal aggression.

R1 KO mice display altered maternal behavior and only moderate differences in aggression. Specifically, these dams spend less time nursing, licking and grooming, and in the nest than wild-types, which may contribute to lower pup weight (Gammie et al., 2007). They also show decreased nest building, eating, and drinking (Gammie et al., 2007; Giardino and Ryabinin, 2013). Although these dams do show a moderate reduction in the number of attacks, and time spent in aggressive behavior toward an intruder, these differences only emerge with repeated testing and are not present initially (Gammie et al., 2007). The latter study suggests that R1 have a role in maternal behavior, and this role is not specific to aggression.

In contrast to the effects of CRF and urocortins to reduce maternal aggression in mice (Gammie et al., 2004; D'Anna and Gammie, 2009), CRF administered i.c.v. inhibits maternal behavior and increases infanticide in virgin female rats (Pedersen et al., 1991). These effects are blunted in females with prior experience with pups (Pedersen et al., 1991), which suggests that repeated exposure to pups may lead to alterations in the CRF system. This is supported by a study showing that repeated experience with pups increases R1 density in the CeA, but not PVN, of female juvenile rats (Rees et al., 2008).

Studies of socially monogamous species also support a role for the CRF system in alloparental and paternal behaviors. Peripheral administration of Ucn2 increases alloparental care in both female and male prairie voles at a dose that does not affect either elevated plus maze or forced swim test behaviors (Samuel et al., 2008). In paternal California mice, fatherhood is associated with reduced CRF-ir in the PVN, but not the CeA or BNST (Chauke et al., 2012). This is specific to fathers, as expectant fathers do not differ from virgin males in any region examined. It is safe to assume that both CRF R1 and R2-acting peptides are involved in regulation of parental behaviors, although the specific contribution of each merits attention.

## Affiliative, attachment, and sexual behaviors

The primary focus of CRF research on social behavior has been on the deleterious effects of social stress on the CRF system, and less is known about the protective effects the social environment may have against stressors via CRF and urocortins (but see Babygirija et al., 2012). However, there are insights on the role of CRF-like peptides in prosocial behavior from the vole and bird literature. Species differences in peptide systems often reflect social organization, with patterns of peptides or their receptors differing in a region-specific manner in species with differing social systems and behavior. Such differences have been observed in the CRF system, and are thought to contribute to species-specific social behavior. The most thorough characterizations have been made in voles, between monogamous and promiscuous vole species. While CRF and Ucn1 are generally conserved across vole species, there is higher CRF mRNA and protein in the PVN, median raphe and BNST and higher Ucn1 mRNA and protein in the Edinger–Westphal nucleus of monogamous species (Lim et al., 2006). Species differences are also observed in CRF receptors, as there is greater R2 binding in the striatum and less R1 binding in the nucleus accumbens shell, olfactory bulb, and superior colliculus of monogamous species (Lim et al., 2005).

The general pattern of CRF receptor distribution in songbirds is similar to mammals. Goodson et al. (2006) have also characterized species differences in CRF receptor binding that reflect sociality. Comparisons were made between five species of estrildid finches and waxbills: one modestly gregarious species, two colonial species, and two territorial species. CRF receptor binding of territorial species differed from modestly gregarious and colonial species in a number of hypothalamic, septal, and forebrain areas (Goodson et al., 2006). In the posterior septum the moderately gregarious Andolan blue waxbill shows receptor binding similar to territorial species, not colonial species. It is not known how these species differences are reflected for the R1 and R2 subtypes, as receptor binding was examined using radiolabeled sauvagine, which can bind non-selectively to CRF receptors. There are two particularly compelling aspects of this study. First, the species selected all shared similar mating, biparental, seasonal, and territorial behavioral characteristics, which minimizes confounds that may be observed in other species comparisons, such as voles. Secondly, the species differences in sociality were independently evolved, suggesting that convergent evolution of CRF receptor distribution is important for social behavior.

The CRF system also plays a role in pair bonding, as administration of CRF directly into the ventricles or nucleus accumbens facilitates partner preference formation in male prairie voles, even at doses that are thought to be too low to increase anxiety (DeVries et al., 2002; Lim et al., 2007). These effects are blocked by co-administration of the either a R1 or R2 antagonist, therefore both receptor subtypes are necessary for pair bond formation (Lim et al., 2007). These findings are particularly interesting as many studies have implicated the CRF system in social memory (described in the next section of the review).

Given that stress inhibits sexual behavior, it is not surprising that the CRF system has also been implicated in sexual behavior. Female white-crowned sparrows (*Zonotrichia leucophrys*) show stress-induced suppression of reproductive behavior during the breeding season. Exogenous CRF (i.c.v.) also inhibits reproductive behavior in this species (Maney and Wingfield, 1998). It is likely that these effects of CRF on behavior are centrally mediated, as this study used a dose of CRF (25 ng) that does not activate the HPA axis, and birds were estradiol implanted to maintain elevated sex steroid levels regardless of CRF treatment. A similar inhibition of sex behaviors is seen in both male and female mice that transgenically overexpress CRF in the brain (Heinrichs et al., 1997). These effects are not affected by adrenalectomy, further supporting a role for central CRF R1-acting peptides (CRF and/or Ucn1) in mediating sexual behavior (Heinrichs et al., 1997).

When injected directly into the arcuate-ventromedial area of the hypothalamus, MPOA, or periaqueductal gray of female rats, CRF inhibits lordosis and increases aggressive rejection of male attempts to sniff and mount (Sirinathsinghji et al., 1983; Sirinathsinghji, 1985, 1986). Similarly, i.c.v. CRF suppresses male copulatory behavior in rats (Sirinathsinghji, 1987). Female Syrian hamsters also show reduced lordosis when either Ucn1 or CRF is administered in the lateral ventricles, and this inhibitory effect of CRF is blocked by co-administration of the CRF receptor antagonist astressin, which suggests that these effects are receptor mediated (Jones et al., 2002). Although the role of each receptor subtype in modulating sexual behavior has not been explored, there is some evidence in support of the R1 receptor in primates. Oral administration of the R1 antagonist antalarmin increased masturbation in rhesus macaques (Habib et al., 2000) and potentiates separation-induced sexual behavior in marmosets (French et al., 2007). Clearly, the inhibitory effects of CRF administration on sexual behavior are seen across many species. However, i.c.v. CRF actually promotes sexual activity in female musk shrews (Schiml and Rissman, 2000), again highlighting the importance of species differences in interactions of the CRF system and social behaviors.

## Social memory

Heinrichs (2003) demonstrated bi-directional effects of pharmacological manipulation of the CRF system on social memory. In these experiments, adult female rats were given social recognition tests following i.c.v. injections of either the general CRF receptor antagonist D-Phe CRF (12–41) or the CRFBP ligand inhibitor r/h CRF (6–33). In this social recognition test, rats are briefly exposed to a novel juvenile conspecific. After a short delay, rats are re-introduced to the same juvenile. A reduction in time spent investigating the juvenile on the second exposure compared to the first is indicative of a social memory for that individual. Each the CRF receptor antagonist and CRFBP inhibitor dose-dependently impaired social memory when the delay between exposures was 30 min (Heinrichs, 2003). Paradoxically, after a 120 min delay, which normally is too long to maintain social recognition in this task, r/h CRF-treated animals displayed social recognition at both doses tested. Importantly, none of these manipulations affected exploration of the juvenile on first exposure, and therefore did not affect anxiety or SI, but only later recognition.

Social investigation and recognition are increased in mice centrally over-expressing CRF (CRF-OE; Kasahara et al., 2011). Mice were habituated to the same juvenile over four serial sessions. On the first habituation, the CRF-OE mice engaged in more social investigation of the juvenile. This contrasts with the effects of pharmacological treatment with CRF to reduce SI. Over the next three sessions, CRF-OE animals showed the same investigation and habituation as wild-types. Ten minutes following the last habituation session, each mouse was exposed to the familiar and a novel juvenile conspecific. Both groups selectively investigated the novel juvenile, indicative of normal short-term memory. However, when tested again 24 h later, only the CRF-OE mice retained social memory (Kasahara et al., 2011).

Both of these studies implicate the CRF system in social recognition, although it remains unclear if this is a general effect on memory, or if it is specific to social stimuli. Evidence from Ucn3 KO mice has identified a disassociation between social and objection recognition (Deussing et al., 2010). Mice lacking Ucn3 demonstrated slower extinction of social memory, but no difference in a novel object recognition task, social odor discrimination, or the SI test. These KOs also demonstrate similar elevated plus maze, forced swim test, acoustic startle, and prepulse inhibition behavior to their wild-type counterparts. In agreement with these findings, mice lacking the CRF R2 receptor also show improved social memory, but Ucn2 KO's have unaltered social memory. This study clearly demonstrates a specific role of Ucn3 on social memory via R2 receptors (Deussing et al., 2010).

## Conclusions and future directions

As reviewed here, the CRF system is both highly sensitive to the social environment and is implicated in the regulation of a broad array of social behaviors. This interaction between social environment and the CRF system is dynamic. It is developmentally sensitive, species-specific, and often sex-specific (Lee et al., 2008; Deussing et al., 2010). The specificity of contribution of CRF R1 vs. R2 receptors to particular social behaviors is in agreement with the general tendency of involvement of various components of the CRF system in different bodily functions across evolution. Perhaps this specificity is a reflection of differential need to control different behavioral states of the organism across behaviorally-different taxa (for example, parental state in monogamous vs. promiscuous species, aggression in territorial vs. communal species, etc).

While it is undeniable that many of the social functions of the CRF system are likely indirect due to stress or anxiety, there are studies indicating differential sensitivity or specificity for social factors and the CRF system. Perhaps the most compelling evidence of this comes from comparative research on the CRF system in closely related species with divergent social behaviors (Lim et al., 2005; Goodson et al., 2006). Further characterization of how the CRF system is involved in species-specific social behavior is a major frontier in understanding the evolution and neurobiology of sociality.

With the rapid progress of the social neuroscience field and in understanding the complex structure of the CRF system, the next challenge is in figuring out the exact contribution of individual components of this system to specific facets of social behaviors. There is substantial evidence that R2 receptors are more responsive to several types of social behaviors. Yet, the original papers frequently only discuss CRF as the ligand potentially mediating this effect. Often this is done without performing parallel studies on the primary ligands of the R2 receptor: the urocortins. Sometimes, to confirm involvement of CRF in a behavior regulated by a CRF antagonist, CRF immunoreactivity or mRNA levels are examined, and changes are taken as an evidence for CRF's involvement. However, expression of CRF and Ucns are co-dependent (for example, CRF KOs have higher levels of Ucn1, and CRF overexpressors have lower levels of Ucn1). Therefore, in the absence of Ucn studies, regulation of CRF is only a suggestive evidence for its involvement over involvement of Ucns (Weninger et al., 2000; Kozicz et al., 2004). While there is direct evidence for the Ucns role in some social behaviors, such as parental behavior and social memory, the Ucns have been largely neglected in many areas areas, including social stress and SI. Providing a complete characterization of the neurocircuity that includes all elements of the CRF system will be invaluable in our understanding of the neurobiology of social behavior and relationships.

### Conflict of interest statement

The authors declare that the research was conducted in the absence of any commercial or financial relationships that could be construed as a potential conflict of interest.

## Abbreviations

BLA: basolateral amygdala
BNST: bed nucleus of the stria terminalis
CeA: central amygdala
CRF: corticotrophin-releasing factor
CSD: chronic social defeat
DRN: dorsal raphe nucleus
HPA: hypothalamic-pituitary-adrenal
HPI: hypothalamic-pituitary-interrenal
-ir: immunoreactivity
KO: knock out
LC/PBN: locus coruleus/parabrachial nucleus
MS: maternal separation
PND: postnatal day
PVN: paraventricular nucleus of the hypothalamus
SDS: social defeat stress
SHRP: stress hyporesponsive period
SI: social interaction
SNP: single nucleotide polymorphism
Ucn: urocortin
VBS: visible burrow system.
